# Bacterial prevalence and inflammatory changes in positive blood culture in community-acquired neonatal sepsis in Jordan

**DOI:** 10.1016/j.ijregi.2025.100756

**Published:** 2025-09-11

**Authors:** Abedulrhman S. Abdelfattah, Hamzeh Al-Momani, Ala'a Al-ma'aiteh, Tamara Kufoof, Amjad Tarawneh, Ahmad Alhroob, Hossam AlNoaimi, Hussein Abu Qaoud, Yazan Alshra’ah, Yara Alhazaimeh, Rahaf Alshorman, Mohammad AlMshagba, Omar Alkhaldi, Ruba Alfuqaha, Mohamadrasoul Alrashaideh

**Affiliations:** 1 Department of Pediatrics, Faculty of Medicine, The Hashemite University, Zarqa, Jordan; 2 Department of Pediatrics, Faculty of Medicine, Mu’tah University, Karak, Jordan; 3 Department of Pediatrics, Al-Bashir Governmental Hospital, Ministry of Health, Amman, Jordan; 4 Faculty of Medicine, The Hashemite University, Zarqa, Jordan

**Keywords:** Community-acquired, Neonatal sepsis, Bacterial prevalence, Inflammatory change

## Abstract

**Objectives:**

This study aimed to identify the most common causative microorganisms of community-acquired neonatal sepsis and assess the diagnostic performance of C-reactive protein (CRP) and complete blood count as predictors for neonatal sepsis.

**Methods:**

This retrospective study examined 359 community-acquired septic neonates in Jordan from 2016 to 2022. Blood cultures, complete blood count, and CRP levels were collected.

**Results:**

Among 359 neonates with community-acquired sepsis, the mean age at presentation was 8.8 days; 61.6% were male, and 55.4% were full-term. Fever (54.6%) and respiratory distress (28.4%) were the most frequent presenting symptoms. Gram-positive and Gram-negative organisms were nearly equal (50.4% vs 49.6%), with *Escherichia coli* (17%) and *Streptococcus viridans* (16.4%) being the most common isolates. Mortality occurred in 11.1% of cases. Diagnostic analysis showed variable utility of inflammatory markers. CRP demonstrated limited discrimination with an area under the curve (AUC) of 0.64, moderate sensitivity (67.4%) and specificity (60.1%). White blood cell abnormalities showed weaker accuracy with AUC values of 0.54 for leukocytosis and 0.43 for leukopenia, with fair specificity (54.5%) but low sensitivity (39.8%). Neutrophil count abnormalities provided better diagnostic utility with AUC values of 0.63 for neutropenia and 0.57 for neutrophilia, yielding moderate sensitivity (64.1%) and specificity (51.1%). Thrombocytopenia (AUC 0.46) and thrombocytosis (AUC 0.41) demonstrated the lowest diagnostic performance, with low sensitivity and moderate specificity being 28.2% and 61.2%, respectively.

**Conclusions:**

Early diagnosis and testing for inflammatory markers are essential for detecting community-acquired neonatal sepsis. However, because each test has limited sensitivity and specificity, combining these markers is necessary to improve diagnostic accuracy.

## Highlights

• Neonatal sepsis is hard to diagnose; inflammatory markers have variable accuracy.
• This study shows bacterial causes of neonatal sepsis in Jordan; marker combos help.
• Using combined inflammatory markers aids early diagnosis in low-resource settings.

## Graphical Abstract

**Figure:**
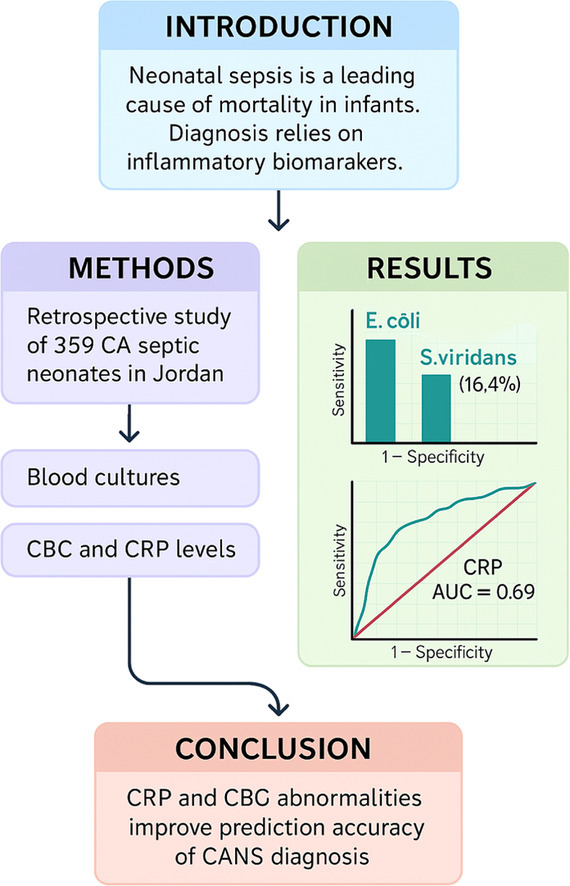

## Introduction

Sepsis is a leading cause of neonatal morbidity and mortality, with most cases occurring in middle-income nations, showing an incidence of 2824 per 100,000 live births and a 17.6% death rate [1]. Each year, one million newborns die from bacterial infections; 99% occur in developing nations, with over half linked to community settings or home deliveries [2]. Neonatal sepsis (NS) is defined as systemic clinical and hemodynamic disturbances caused by pathogenic microorganisms in sterile body fluids during the first month of life [3].

Based on the timing of onset, NS is categorized into early-onset sepsis. This type occurs during the first 3 days of life [4] and is mainly caused by bacterial transmission from the mother’s genital tract either before or during delivery. In contrast, late-onset sepsis (LOS) arises after the first 3 days of birth, maybe either hospital-acquired or community-acquired NS (CANS) [5]. LOS, including both types, is mostly caused by Gram-positive strains, commonly coagulase-negative *Staphylococci* [6].

Gestational age and birth weight are the most frequently reported factors that contribute to LOS and increase the likelihood of its development [7]. Etiological studies have linked LOS in neonates to a variety of pathogenic organisms. The frequently identified Gram-positive bacteria detected among septic neonates are *Streptococcus viridans* and *Staphylococcus aureus*, whereas *Escherichia coli* and *Klebsiella pneumoniae* are the most commonly identified Gram-negative bacteria [8].

The clinical presentation of NS is often non-specific. Symptoms such as hypothermia or fever, jaundice, respiratory distress, lethargy, and poor feeding can hinder early detection and may lead to excessive use of antibiotics [9]. Blood cultures are crucial for diagnosis; however, they take at least 24 hours to yield results, and a negative blood culture does not exclude the diagnosis [10].

The diagnosis mainly relies on positive culture results. Hematological markers in a complete blood count (CBC), such as leukocytosis or leukopenia, neutrophilia or neutropenia, thrombocytopenia, and the immature neutrophil to total neutrophil ratio, along with elevated inflammatory markers like C-reactive protein (CRP) and procalcitonin, as well as other new markers, aid in diagnosing sepsis [11].

Few retrospective studies have assessed NS or CANS in Jordan, with limited data on incidence and outcomes [[12], [13], [14]]. In 2020, the World Health Organization called for more substantial evidence on sepsis burden in low- and middle-income countries [15]. This study, therefore, aimed to identify common causative microorganisms of CANS and assess CRP and CBC as diagnostic predictors for NS.

## Patients and methods

### Study design and setting

This retrospective study investigated CANS among infants aged 3-28 days old with positive blood cultures, who were admitted to the neonatology and pediatric departments of Al-Zarqa Governmental Hospital and Al-Bashir Governmental Hospital, between 2016 and 2022.

### Study sample

A total of 359 neonates were included, as determined by statistical criteria. The sample size calculation utilized a two-sided test with an α value of 0.05 and a β value of 0.2.

### Inclusion and exclusion criteria

Inclusion criteria comprised neonates diagnosed with CANS (aged 3-28 days) admitted to the pediatric ward or neonatal intensive care unit (NICU) who showed symptoms or signs of sepsis after being discharged from the hospital following delivery. Exclusion criteria included neonates presenting sepsis symptoms within the first 3 days of life, as well as cases where microorganisms were identified as contaminants (e.g., coagulase-negative staphylococci [CONS]).

CONS cases were excluded due to the significant variability in how they are treated, making it hard to determine if there was an actual infection. The available medical records did not consistently provide evidence to confirm CONS as a definitive pathogen. To avoid overestimating CONS-related sepsis and to ensure diagnostic accuracy, the analysis was limited to organisms with well-established pathogenic roles.

### Ethical considerations

The study was approved by the Faculty of Medicine at Hashemite University in Jordan (IRB No. 19/4/2022/2023). No consent was required because the study was retrospective in nature.

### Data collection and statistical analysis

All demographic, clinical, and laboratory data were collected from the medical records of the hospitals mentioned above. The data included sex, age at presentation, gestational age, prematurity, birth weight (kg), mode of delivery, history of prolonged rupture of membranes, maternal infection history, CRP levels, white blood cell (WBC) count, neutrophil percentage, platelet count, and absolute neutrophil count (ANC). The symptoms were documented in the emergency room or the NICU by the pediatric physician or neonatologist.

Microorganisms were identified using conventional blood culture methods and the VITEK 2 Compact system (Biomerieux, Marcy l’Étoile, France). Two milliliters of blood were drawn aseptically from different venipuncture sites to confirm the diagnosis of sepsis by isolating the causative agent from the blood culture [16]. Positive blood cultures were identified if only one bacterium was observed growing [17]. In addition, on admission (day 1), laboratory tests and cultures were conducted on all septic newborns before starting antibiotic therapy.

According to hospital protocols, standard laboratory tests, including a CBC and serum CRP levels, were performed upon admission, and samples were collected from peripheral vascular tissue. For all tests, 500 µl of blood drawn in microvials was adequate.

CBC analysis was performed using Sysmex Corporation (Wakinohama-kaigandori, Chuo-ku, Kobe, Japan). Cobas® 6000 (Roche, HITACHI, Germany) was used to determine serum CRP levels according to the manufacturer's instructions.

Data analysis included both descriptive and inferential statistics. Continuous variables, such as age, gestational age, and birth weight, were reported as means ± standard deviations. Categorical variables, including sex, delivery mode, and bacterial type, were presented as frequencies and percentages. Group comparisons were performed using the Chi-square test for categorical variables and independent *t*-tests for continuous variables, with significance set at *P* <0.05. The diagnostic performance of inflammatory markers (CRP, WBC, ANC, and platelets) was evaluated by calculating sensitivity, specificity, positive predictive value (PPV), and negative predictive value (NPV). Receiver operating characteristic curve analysis, including the area under the curve (AUC) and 95% confidence intervals (CIs), was used to assess the discriminatory ability. Statistical analysis conducted using SPSS version 26.

### Definitions

CA sepsis is a severe bacterial infection that develops outside of a hospital or health care setting, such as at home or in a public place. It is characterized by the body’s systemic response to infection, which can lead to life-threatening complications if not diagnosed and treated promptly [18].

According to the study laboratory guidelines, elevated CRP was defined as levels greater than 5 mg/dl. Leukocytosis (WBC >20,000/mm³) and leukopenia (WBC <5000/mm³) were classified as abnormal hematologic findings indicative of infection [[19], [20], [21]].

Neutropenia is defined as decreased ANC and occurs when neutrophil levels fall below 1500 cells/ul. Conversely, neutrophilia is defined as increased ANC and occurs when neutrophil levels rise above 14,500 cells/ul. ANC is calculated by multiplying the WBC count by the total percentage of neutrophils [22].

Thrombocytosis was defined as a platelet count above 450,000, whereas thrombocytopenia was defined as a platelet count below 150,000 [23].

## Results

### General characteristics of the studied neonates

The current study included 359 neonates who were diagnosed with CANS and had positive blood cultures. The mean age at presentation of the included neonates was 8.84 ± 8.213 days, ranging from 3-28 days. More than half of the studied neonates were males (61.6%). The mean gestational age was 36.125 ± 2.36 weeks. More than half of the studied patients were full-term (55.4%), with a mean birth weight of 2.7 ± 0.626 kg. The most common birth delivery among the studied neonates was natural vaginal delivery (77.7%). Prolonged rupture of the membrane occurred among 2.8% of the studied neonates. Only 19.2% of the study neonates had a history of maternal infection. All demographic and baseline characteristic data differed significantly (*P* <0.001) (Table 1).

**Table 1:** Demographic and baseline characteristics of the studied patients.

Variable	Parameter	Statistics	*P*-value
Sex, n (%)	Male ➢	221 (61.6%)	<0.001^a^
	Female ➢	138 (38.4%)	
Age at presentation (days)	Mean ± SD	8.84 ± 8.213	<0.001^a^
	Median (Min-Max)	3 (3-28)	
Gestational age (weeks)	Mean ± SD	36.125 ± 2.36	<0.001^a^
	Median (Min-Max)	38 (31-40)	
Prematurity, n (%)	Full term ➢	199 (55.4%)	0.04^b^
	Preterm ➢	160 (44.6%)	
Birth weight (kg)	Mean ± SD	2.7 ± 0.626	<0.001^a^
	Median (Min-Max)	2.57 (1.8-4.9)	
Mode of delivery, n (%)	Natural vaginal delivery ➢	279 (77.7%)	<0.001^a^
	Cesarean ➢	80 (22.3%)	
History of prolonged rupture of membrane, n (%)	Yes ➢	10 (2.8%)	<0.001^a^
	No ➢	349 (97.2%)	
History of maternal infection, n (%)	Yes ➢	69 (19.2%)	<0.001^a^
	No ➢	290 (80.8%)	

a p-value significant <0.01;

b p-value significant <0.05.

### Symptoms of sepsis among the studied neonates

The most common symptom of sepsis among the studied patients was a fever, reported in 54.59% of patients, followed by respiratory distress, which was observed in 28.4% of the neonates. Other symptoms, such as poor feeding, hypoactivity, jaundice, and vomiting, made up nearly the rest of the presenting symptoms.

### Type of bacteria and Gram staining among the studied neonates

**Figure 1:**
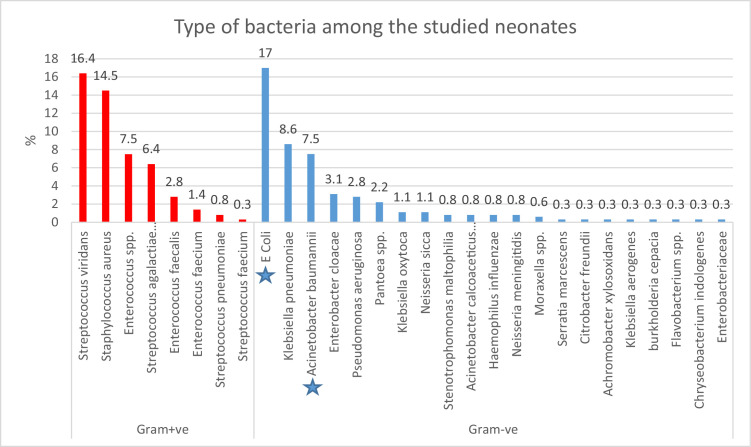
Types of bacteria among the studied patients; star represent the most common causes of sepsis mortality.

Gram-positive and Gram-negative bacteria were present in 50.4% and 49.6% of the studied neonates, respectively. The most common organisms detected among all bacterial causes in these septic neonates were *E. coli* (17%) and *S. viridans* (16.4%). Among the Gram-positive bacteria, *S. viridans* was the most common, present in 16% of patients, followed by *S. aureus* (14.5%). The most common Gram-negative bacteria were *E. coli* (17%) and *K. pneumonia* (14.5%), making them the most prevalent bacteria among the studied patients. *Acinetobacter baumannii* and *E. coli* were the most prevalent bacterial causes among the non-survivors (Figure 1).

### Inflammatory markers according to survival rate among community-acquired septic neonates

Out of the 359 studied neonates, 40 patients (11.1%) died. Among the studied neonates, significant differences in inflammatory markers were observed between survivors and non-survivors. CRP positivity was present in 72.7% of all septic neonates; however, it was much lower in non-survivors (35%) compared with survivors (77.4%, *P* <0.001). Leukopenia was also more frequent among those who died (30%) than among survivors (9.1%), whereas leukocytosis was more common in survivors (32.9%, *P* <0.001). Platelet counts showed a similar pattern, with thrombocytopenia present in nearly half of the non-survivors (47.5%) compared with only 24.8% of survivors (*P* = 0.008). Neutropenia was also more prevalent among non-survivors (60%) than survivors (39.2%, *P* = 0.038). These findings suggest that leukopenia, thrombocytopenia, and neutropenia were associated with poorer outcomes in CANS (Table 2).

**Table 2:** Inflammatory markers according to survival rate among community-acquired septic neonates.

Variable	Parameter	Died (n = 40)	Survived (n = 319)	Test value	*P*-value
C-reactive protein (mg/dl)	Mean ± SD	12.99 ± 32.02	15.06 ± 23.95	32.241	<0.001^a^
	➢ Elevated (72.7%)	14 (35%)	247 (77.4%)		
	➢ Normal (27.3)	26 (65%)	72 (22.6%)		
White blood cell (×10^9^/l)	Mean ± SD	12.53 ± 11.05	15.2 ± 8.19	16.5	<0.001^a^
	➢ Normal (57.4%)	21 (52.5%)	185 (58%)		
	➢ Leukopenia (11.4%)	12 (30%)	29 (9.1%)		
	➢ Leukocytosis (31.2%)	7 (17.5%)	105 (32.9%)		
Absolute neutrophil count (*cells per microliter)*	Mean ± SD	6488.65 ± 9495.7	7603.03 ± 7895.5	6.55	0.038^a^
	➢ Normal (36.5%)	11 (27.5%)	120 (37.6%)		
	➢ Neutropenia (41.5%)	24 (60%)	125 (39.2%)		
	➢ Neutrophilia (22%)	5 (12.5%)	74 (23.2%)		
Platelets (×10^9^/l)	Mean ± SD	169.55 ± 108.15	251.59 ± 131.13	9.55	0.008^a^
	➢ Normal (66.6%)	20 (50%)	219 (68.7%)		
	➢ Thrombocytopenia (27.3%)	19 (47.5%)	79 (24.8%)		
	➢ Thrombocytosis (6.1%)	1 (2.5%)	21 (6.6%)		

a p-value significant <0.05.

### Diagnostic potential of inflammatory marker levels in relation to community-acquired septic neonates at admission

**Figure 2:**
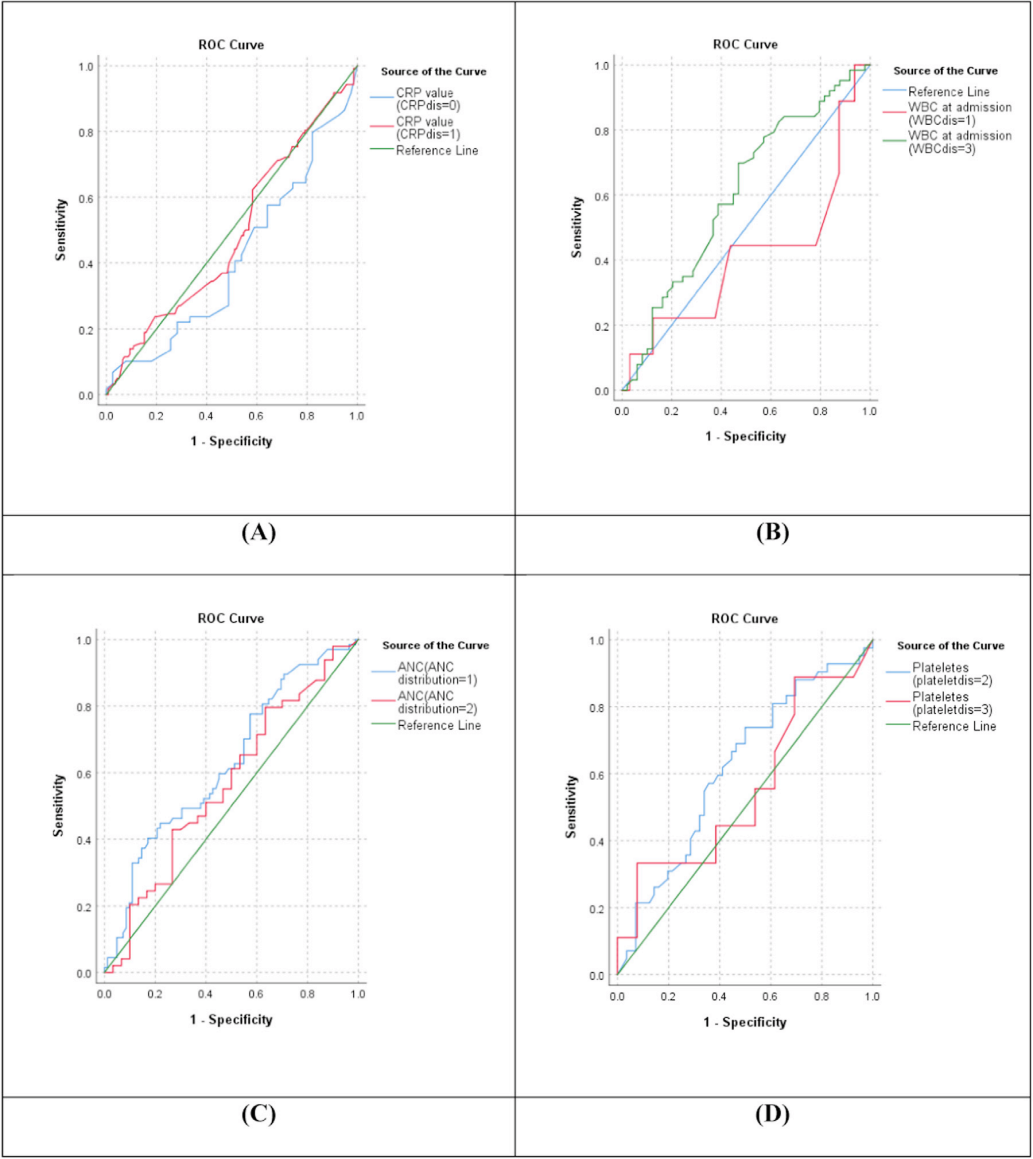
ROC curves of inflammatory marker levels among community-acquired septic neonates. (a) ROC curve for CRP (0 for negative and 1 for elevated), (b) ROC curve for WBC (1 for leukopenia, 3 for leukocytosis), (c) ROC curve for ANC level (1 for neutropenia and 2 for neutrophilia), (d) ROC curve for platelet count (2 for thrombocytopenia, 3 for thrombocytosis).

ANC, absolute neutrophil count; CRP, C-reactive protein; ROC, receiver operating characteristic curve; WBC, white blood cell.

The receiver operating characteristic curve analysis showed that CRP, WBC, ANC, and platelet levels upon admission produced variable AUC for culture-positive CANS detection as follows: elevated CRP (AUC = 0.64 [95% CI 0.52-0.76], *P* = 0.01); regarding WBC abnormalities, leukocytosis showed AUC = 0.604 (95% CI 0.0.503-0.705, *P* = 0.000), whereas leukopenia had less AUC (AUC = 0.41 [95% CI 0.32-0.5], *P* = 0.01). ANC abnormalities in general showed better AUC, for neutrophilia AUC = 0.565 (95% CI 0.52-0.61, *P* = 0.01), whereas neutropenia had better AUC (AUC = 0.629 [95% CI 0.547-0.711], *P* = 0.000). Finally, platelet abnormalities with thrombocytopenia had the better prediction of infection with AUC = 0.618 (95% CI 0.550-0.686, *P* = 0.000) (Figure 2).

The sensitivity, specificity, PPV, and NPV of elevated CRP were 67.4%, 60.2%, 63.2%, and 64.5%, respectively. In contrast, the sensitivity, specificity, PPV, and NPV of WBC abnormality were 39.8%, 54.5%, 47.05%, and 47.1%, respectively. In addition, combining neutropenia and neutrophilia increased the sensitivity, specificity, PPV, and NPV to 64.1%, 51.1%, 64.8%, and 50.4%, respectively. Combining both thrombocytopenia and thrombocytosis improved the sensitivity, specificity, PPV, and NPV of platelet abnormalities to 28.2%, 61.2%, 42.5%, and 45.6%, respectively (Table 3).

**Table 3:** Diagnostic values of inflammatory markers levels upon admission among community-acquired septic neonates.

	True positive	True negative	False positive	False negative	Positive predictive value	Negative predictive value	Sensitivity	Specificity	Area under the curve
C-reactive protein positive	122	107	71	59	63.2	64.5	67.4	60.2	0.64
Leukopenia	9	146	32	172	22	90.9	5	82	0.41
Leukocytosis	63	129	49	118	56.3	52.2	34.8	72.5	0.604
White blood cell abnormality	72	97	81	109	47.05	47.1	39.8	54.5	
Thrombocytopenia	42	122	56	139	42.9	46.7	23.2	68.5	0.618
Thrombocytosis	9	165	13	172	40.9	48.9	5	93	0.556
Platelets abnormality	51	109	69	130	42.5	45.6	28.2	61.2	
Neutropenia	67	96	82	114	45	45.7	37	54	0.629
Neutrophilia	49	148	30	132	62	52.8	27.1	83.2	0.565
Absolute neutrophil count abnormality	116	66	63	65	64.8	50.4	64.1	51.1	

## Discussion

One of the most significant challenges neonatologists and pediatricians encounter in the NICU is the early detection of NS due to its vague clinical presentation, long diagnostic times, and high fatality rate [24].

### Bacterial pathogens in CANS

Gram-positive and Gram-negative bacteria were detected in 50.4% and 49.6% of the studied neonates, respectively. The most frequently identified organisms were *E. coli* (17%) and *S. viridans* (16.4%), followed by *S. aureus* and *K. pneumoniae*.

Research by Downie et al. [25] reported that in CA septic neonates, sepsis is most commonly caused by *S. aureus, Klebsiella species*, and *E. coli*, with *E. coli* accounting for 55% of culture-positive sepsis cases. In Zaidi et al.’s [26] study, *Klebsiella species* were the most common cause of CANS among developing nations (25%), followed by *S. aureus* (18%) and *E. coli* (15%). They also found that *S. aureus* (14%), Group B *Streptococcus* (12%), *Streptococcus pneumoniae* (12%), and nontyphoidal *Salmonella species* (13%) were commonly observed during the early neonatal period [26]. Moreover, Gram-negative bacteria were associated with worse outcomes, a finding confirmed in the present study and consistent with previous research [25,26].

### CRP as an inflammatory marker

Elevated CRP levels were observed in 72.7% of all patients, which is higher than the rates reported by Sorsa (2018) and Bhat and Baby (2011), who found elevated CRP in 45% and 69% of CANS patients, respectively [20,27]. A key finding in our study was that CRP levels at diagnosis were not significantly associated with mortality in culture-positive CANS, underscoring the need for a thorough assessment of the neonates presented with signs and symptoms of sepsis.

In contrast, several studies have identified CRP as a strong predictor of newborn sepsis outcomes. However, due to its physiological rise after delivery, CRP exhibits limited specificity [28,29].

In this study, elevated CRP demonstrated the highest sensitivity and specificity among the inflammatory markers assessed, with sensitivity, specificity, PPV, and NPV of elevated CRP being 67.4%, 60.2%, 63.2%, and 64.5%, respectively. Still, these values did reach those reported in other studies, which documented better diagnostic performance for CRP in NS [29,30].

### White blood cell abnormalities

Abnormal WBC counts, including leukopenia (<5000) and leukocytosis (>20,000), were observed in 11.4% and 31.2% of CA septic neonates, respectively. The combined sensitivity, specificity, PPV, and NPV for WBC abnormalities were 39.8%, 54.5%, 47.05%, and 47.1%, respectively, indicating that WBC abnormalities alone have limited diagnostic accuracy. Although leukocytosis showed better discriminatory power than leukopenia (AUC 0.604 vs 0.41), leukopenia was significantly associated with mortality in non-survivors.

These findings contrast with previous research, which showed that leukopenia is generally a more reliable indicator of sepsis [31]. The review by Sharma et al., which reported that leukopenia (WBC count <5000/mm³) has high specificity (91%) but lower sensitivity (29%) when used to identify newborn sepsis [32]. However, leukocytosis has been frequently investigated and found to have substantial predictive value for newborn sepsis in several studies [20,29,33].

### Neutrophil count abnormalities

Neutropenia and neutrophilia were observed in 41.5% and 22% of CA septic neonates, respectively. When both conditions were combined, neutropenia and neutrophilia, the sensitivity, specificity, PPV, and NPV of ANC abnormalities increased to 64.1%, 51.1%, 64.8%, and 50.4%, respectively. Although neutrophil count abnormalities showed better sensitivity and specificity than WBC abnormalities, their diagnostic accuracy remained limited.

These findings align with the study by Bhale et al*.* [34], who found that low ANC revealed lower sensitivity (42.9%), but unlike the current study, the specificity was much higher (99.0%). Among all septic screening criteria, ANC had the highest specificity and a PPV of 97.5% [34]. Neutropenia has greater discriminatory power and predictive value for mortality than neutrophilia (AUC 0.629 vs 0.565), as previous studies have revealed [34]. Conversely, Derbala et al*.* [35] indicated that ANC sensitivity was higher (97.5%) and specificity was lower (36.7%), highlighting the variability of ANC in detecting sepsis across different studies.

### Platelet count abnormalities

Thrombocytopenia and thrombocytosis were found in 27.3% and 6.1% of CA septic patients, respectively. When combining both conditions, the sensitivity, specificity, PPV, and NPV for platelet abnormalities improved to 28.2%, 61.2%, 42.5%, and 45.6%, respectively. Thrombocytopenia often indicates a poor prognosis and is considered a significant marker for sepsis [36]; however, the sensitivity of thrombocytopenia in this study was lower than that reported in Nigerian (56%) [37], Egyptian (56.7%) [35], and Indian (53.09%) [38] studies. On the other hand, the specificity of thrombocytopenia was higher than in Nigerian (56%), Egyptian (56.7%), and Indian (53.09%) studies. These results suggest that thrombocytopenia retains some discriminatory value, as supported by the current research (AUC = 0.618); however, it was significant in predicting adverse outcomes.

### Combined diagnostic indicators

Because no single inflammatory marker demonstrated sufficient sensitivity, specificity, or discriminative power for sepsis, combining CRP, WBC, ANC, and platelet count abnormalities considerably improved diagnostic accuracy compared with using each marker alone. This aligns with studies from Italy and Tanzania, which reported that the presence of two abnormal markers achieved sensitivities of 93-100%, specificity of 83%, PPV of 27%, and NPV of 100% for culture-confirmed sepsis [29,32,39].

### Strengths and limitations of the study

The large cohort size and multicenter design are major strengths, enabling inclusion of diverse clinical and demographic factors. Conducted at two major Jordanian hospitals, which cover most pediatric and neonatal services, the study identified common causative organisms of CANS in a developing-country setting, helping to guide policies aimed at reducing NS.

However, several limitations exist. The absence of a control group limited direct comparisons, and reliance on existing medical records risked missing or incomplete data, thereby constraining the evaluation of CONS as a cause of sepsis. Additional inflammatory markers, such as procalcitonin, were unavailable. Finally, the lack of serial blood sampling (e.g., every 24-48 hours after antibiotics) restricted the monitoring of inflammatory marker changes over time and their link to clinical outcomes.

## Conclusion

Early diagnosis and lab investigation of infection evidence are crucial for the early detection of CANS. The most common organisms identified among all bacterial causes in the studied septic neonates were *E. coli* and *S. viridans*. CRP, WBC, platelets, and ANC used to be inflammatory markers for early detection of CANS in developing countries. However, because each test lacks both sensitivity and specificity, combining these inflammatory markers is necessary to improve diagnostic accuracy and predict outcomes.

## Declaration of competing interest

The authors have no competing interests to declare.

## References

[1] Fleischmann C., Reichert F., Cassini A., Horner R., Harder T., Markwart R. (2021). Global incidence and mortality of neonatal sepsis: a systematic review and meta-analysis. Arch Dis Child.

[2] Waters D., Jawad I., Ahmad A., Lukšić I., Nair H., Zgaga L. (2011). Aetiology of community-acquired neonatal sepsis in low and middle income countries. J Glob Health.

[3] Al Bakoush F.B., Azab A.E., Yahya R.A. (2023). Neonatal sepsis: insight into incidence, classification, risk factors, causative organisms, pathophysiology, prognosis, clinical manifestations, complications, systemic examination, and treatment. South Asian Res J App Med Sci.

[4] Simonsen K.A., AL Anderson-Berry, Delair S.F., Davies HD. (2014). Earlyonset neonatal sepsis. Clin Microbiol Rev.

[5] Amer Y.S., Shaiba L.A., Hadid A., Anabrees J., Almehery A., AAssiri M. (2022). Quality assessment of clinical practice guidelines for neonatal sepsis using the Appraisal of Guidelines for Research and Evaluation (AGREE) II instrument: a systematic review of neonatal guidelines. Front Pediatr.

[6] Zea-Vera A., Ochoa T.J. (2015). Challenges in the diagnosis and management of neonatal sepsis. J Trop Pediatr.

[7] Murthy S., Godinho M.A., Guddattu V., Lewis L.E., Nair NS. (2019). Risk factors of neonatal sepsis in India: a systematic review and meta-analysis. PLoS One.

[8] Kim T.W., Lee S.U., Park B., Jeon K., Park S., Suh G.Y. (2023). Clinical effects of bacteremia in sepsis patients with community-acquired pneumonia. BMC Infect Dis.

[9] Kasmire K.E., Vega C., Bennett N.J., Laurich VM. (2021). Hypothermia: a sign of sepsis in young infants in the emergency department?. Pediatr Emerg Care.

[10] Procianoy R.S., Silveira RC. (2020). The challenges of neonatal sepsis management. J Pediatr (Rio J).

[11] Boscarino G., Migliorino R., Carbone G., Davino G., Dell’Orto V.G., Perrone S. (2023). Biomarkers of neonatal sepsis: where we are and where we are going. Antibiotics (Basel).

[12] Khassawneh M., Khader Y., Abuqtaish N. (2009). Clinical features of neonatal sepsis caused by resistant Gram-negative bacteria. Pediatr Int.

[13] Younis NS. (2011). Neonatal sepsis in Jordan: bacterial isolates and antibiotic susceptibility patterns. Rawal Med J.

[14] Yusef D., Shalakhti T., Awad S., Algharaibeh H.A., Khasawneh W. (2018). Clinical characteristics and epidemiology of sepsis in the neonatal intensive care unit in the era of multidrug-resistant organisms: a retrospective review. Pediatr Neonatol.

[15] World Health Organization (2020).

[16] Nagabhushan S. (2013). Study of aerobic bacterial profile of bloodstream infections and antibiotic susceptibility pattern of the isolates.

[17] Goldstein B., Giroir B., Randolph A., International Pediatric Sepsis Consensus Conference (2005). International pediatric sepsis consensus conference: definitions for sepsis and organ dysfunction in pediatrics. Pediatr Crit Care Med.

[18] Giannoni E., Agyeman P.K., Stocker M., Posfay-Barbe K.M., Heininger U., Spycher B.D. (2018). Neonatal sepsis of early onset, and hospital-acquired and community-acquired late onset: a prospective population-based cohort study. J Pediatr.

[19] Remington J.S., Wilson C.B., Nizet V., Klein J.O., Maldonado Y. (2011). Infectious diseases of the fetus and newborn.

[20] Sorsa A. (2018). Diagnostic significance of white blood cell count and C-reactive protein in neonatal sepsis; Asella Referral Hospital, South East Ethiopia. Open Microbiol J.

[21] Long S.S., Kimberlin D.W., Brady M.T., Jackson M.A., Long S.S. (2012). Principles and practice of pediatric infectious diseases.

[22] Doig K., Thompson LA. (2017). A methodical approach to interpreting the white blood cell parameters of the complete blood count. Clin Lab Sci.

[23] Gonzalez B.E., Mercado C.K., Johnson L., Brodsky N.L., Bhandari V. (2003). Early markers of late-onset sepsis in premature neonates: clinical, hematological, and cytokine profile. J Perinatol.

[24] Nyenga A.M., Mukuku O., Wembonyama SO. (2021). Neonatal sepsis: a review of the literature. Theory Clin Pract Pediatr.

[25] Downie L., Armiento R., Subhi R., Kelly J., Clifford V., Duke T. (2013). Community-acquired neonatal and infant sepsis in developing countries: efficacy of WHO’s currently recommended antibiotics—systematic review and meta-analysis. Arch Dis Child.

[26] Zaidi A.K., Thaver D., Ali S.A., Khan TA. (2009). Pathogens associated with sepsis in newborns and young infants in developing countries. Pediatr Infect Dis J.

[27] Bhat Y.R., Baby LP. (2011). Early onset of neonatal sepsis: analysis of the risk factors and the bacterial isolates by using the BacT alert system. J Clin Diagn Res.

[28] Sproston N.R., Ashworth JJ. (2018). Role of C-reactive protein at sites of inflammation and infection. Front Immunol.

[29] Shane A.L., Sánchez P.J., Stoll BJ. (2017). Neonatal sepsis. Lancet.

[30] Li X., Li T., Wang J., Feng Y., Ren C., Xu Z. (2021). Clinical value of C-reactive protein/platelet ratio in neonatal sepsis: a cross-sectional study. J Inflamm Res.

[31] Newman T.B., Puopolo K.M., Wi S., Draper D., Escobar GJ. (2010). Interpreting complete blood counts soon after birth in newborns at risk for sepsis. Pediatrics.

[32] Sharma D., Farahbakhsh N., Shastri S., Sharma P. (2018). Biomarkers for diagnosis of neonatal sepsis: a literature review. J Matern Fetal Neonatal Med.

[33] Chiesa C., Pellegrini G., Panero A., Osborn J.F., Signore F., Assumma M. (2003). C-reactive protein, interleukin-6, and procalcitonin in the immediate postnatal period: influence of illness severity, risk status, antenatal and perinatal complications, and infection. Clin Chem.

[34] Bhale C.P., Kale A.V., Kale S.S., Mahajan M.E., Smulay SM. (2016). Utility of sepsis screen in the early diagnosis of neonatal sepsis. Indian J Neonatal Med Res.

[35] Derbala S., Handoka N., Hasan E., ElSayed H. (2017). Performance of the hematological scoring system for early diagnosis of neonatal sepsis in a neonatal intensive care unit of a developing country. Infect Dis Trop Med.

[36] Bhalodia M.J., Hippargi S.B., Patil M. (2017). Role of hematological scoring system in diagnosis of neonatal sepsis. J Clin Neonatol.

[37] Pius S., Bello M., Galadima G.B., Bukar A., Mava Y., Ambe JP. (2016). Clinical features and haematological indices of neonatal septicaemia in poor resource settings. Open J Pediatr.

[38] Shah H., Jha B. (2019). Early diagnosis and evaluation of neonatal septicemia by hematological scoring system. Int J Med Sci Public Health.

[39] Chacha F., Mirambo M.M., Mushi M.F., Kayange N., Zuechner A., Kidenya B.R. (2014). Utility of qualitative C-reactive protein assay and white blood cell counts in the diagnosis of neonatal septicaemia at Bugando Medical Centre, Tanzania. BMC Pediatr.

